# Sex Disparities in Outcome of Patients with Alcohol-Related Liver Cirrhosis within the Eurotransplant Network—A Competing Risk Analysis

**DOI:** 10.3390/jcm11133646

**Published:** 2022-06-24

**Authors:** Stephan Listabarth, Daniel König, Gabriela Berlakovich, Petra Munda, Peter Ferenci, Dagmar Kollmann, Georg Gyöeri, Thomas Waldhoer, Magdalena Groemer, Arjan van Enckevort, Benjamin Vyssoki

**Affiliations:** 1 Clinical Division of Social Psychiatry, Department of Psychiatry and Psychotherapy, Medical University of Vienna, 1090 Vienna, Austria; stephan.listabarth@meduniwien.ac.at (S.L.); daniel.koenig@meduniwien.ac.at (D.K.); magdalena.groemer@meduniwien.ac.at (M.G.); benjamin.vyssoki@meduniwien.ac.at (B.V.); 2 Division of Transplantation, Department of Surgery, Medical University of Vienna, 1090 Vienna, Austria; gabriela.berlakovich@meduniwien.ac.at (G.B.); dagmar.kollmann@meduniwien.ac.at (D.K.); georg.gyoeri@meduniwien.ac.at (G.G.); 3 Division of Gastroenterology and Hepatology, Department of Internal Medicine III, Medical University of Vienna, 1090 Vienna, Austria; petra.munda@meduniwien.ac.at (P.M.); peter.ferenci@meduniwien.ac.at (P.F.); 4 Center for Public Health, Department of Epidemiology, Medical University of Vienna, 1090 Vienna, Austria; 5 Eurotransplant International Foundation, 2301 Leiden, The Netherlands; request@eurotransplant.org

**Keywords:** alcohol-related liver cirrhosis, liver transplantation, gender medicine, outcome research, alcohol use disorder, addiction

## Abstract

Alcohol use disorder (AUD) is one of the most important risk factors for the development of alcohol-related liver cirrhosis (ALC). Importantly, psychiatrists are an integral part of the interdisciplinary care for patients with AUD and ALC. The aim of the current study was to investigate whether sex influences the outcome within this group of patients. For this purpose, data of all registrations for liver transplantations due to ALC within the Eurotransplant region from 2010 to 2019 were analyzed for sex disparities using competing risk models and in-between group comparisons. Relevant sex differences in registration numbers (24.8% female) and investigated outcomes were revealed. Risk ratios for a positive outcome, i.e., transplantation (0.74), and those of adverse outcomes, i.e., removal from waiting list (1.44) and death on waiting list (1.10), indicated a relative disadvantage for female patients with ALC. Further, women listed for liver transplantations were significantly younger than their male counterparts. Notably, sex disparities found in registration and outcome parameters were independent of differences found in the prevalence of AUD and liver transplantations. Further research is necessary to identify the underlying mechanisms and establish strategies to ensure equity and utility in liver transplantations due to ALC.

## 1. Introduction

Alcohol use disorder (AUD) is the most common diagnosis within the group of substance use disorders and—despite strong efforts—prevalence rates worldwide remain high (1.45% for the total population, males only 2.27%) [1]. Even higher prevalence rates have been published for Western Europe (2.35%, males: 3.66%) [2]. Alcohol use disorder causes a relevant burden on the affected individual and represents a significant contributing factor to the global disease burden [1]. Importantly, long-term alcohol use is one of the principal risk factors for ill-health and is associated with numerous health conditions, especially the development of liver diseases [1]. Additionally, alcohol is the most decisive factor for premature death in the age group of 15- to 49-year-olds [3].

Individuals exhibiting high-risk drinking patterns have a significantly increased risk of developing alcohol-related liver disease [4,5], including alcohol-related liver cirrhosis (ALC), a condition with a poor prognosis [6]. As a healthy liver is crucial for maintaining multiple physiological functions (e.g., detoxification, digestion, syntheses of proteins and storage of glycogen), liver damage can have severe consequences. Liver damage by excessive alcohol consumption emerges due to a complex interplay between direct cytotoxic substances accumulating during alcohol metabolism and fibrogenesis occurring as a response mechanism to tissue damage, progressively replacing functional liver tissue with non-functional connective tissue [7]. Thus, a continuum of alcohol-related liver cirrhosis can occur, ranging from fibrosis to steatosis to compensated and, in later stages, decompensated liver cirrhosis. Progression along this continuum is significantly associated with the amount of alcohol consumed [4,5].

Besides alcohol, a variety of other factors are known to cause liver cirrhosis, including obesity, non-alcoholic fatty liver diseases, chronic viral infections, cholestatic diseases, autoimmune diseases and diseases with metal overload (i.e., Morbus Wilson, iron overload) [8]. While improvements in the prevention and treatment of virus-related liver cirrhosis have facilitated decreasing death rates from non-alcohol-related liver cirrhosis, increasing prevalence rates for ALC have caused a surge in liver-cirrhosis-related deaths, particularly in Eastern Europe and Central Asia [9]. While multi-disciplinary interventions primarily aim to reduce alcohol consumption in patients with AUD and complete abstinence in patients with ALC, respectively, in certain stages of these diseases, in which liver damage has reached an irreversible level, further interventions are necessary. Thus, for some patients, a liver transplantation is the only remaining curative treatment, as mortality due to severe complications of ALC is high [6]. In fact, ALC is the most common indication for liver transplantation (LT) in European countries [10,11,12].

Solid-organ transplantations are inherently linked to ethical deliberations, including considerations regarding the selection criteria of potential recipients, specific indications and, most importantly, the prioritization of patients on the waiting list. Transparent organ allocation rules are, therefore, of utmost importance [13,14]. The stated considerations become even more pertinent in the context of organ scarcity and disequilibrium of supply and demand [13,15,16]. In the specific case of ALC-related LT, additional ethical concerns have been raised and discussed controversially since the introduction of LT [17,18,19]. Foremost, the misconception of all-dominant self-responsibility in the onset of alcohol-induced liver cirrhosis persists, even for medical professionals. However, evidence unambiguously points towards the concept of (alcohol) use disorder as a medical condition, rather than being a ‘personal weakness’ or a ‘matter of character’ and patients, therefore, should be treated accordingly [20]. This is equally applicable for patients with AUD and ALC, to whom pharmacological and psychotherapeutic treatment options, including brief interventions and cognitive behavioral therapy, should be offered [21,22].

All mentioned aspects emphasize the necessity for solid ethical guidelines that facilitate adequate access to lifesaving LT, including the prioritization and handling of waiting list metrics. Efforts in this regard have fostered the emergence of numerous policies and regulations regarding the selection of appropriate individuals with alcohol-induced liver cirrhosis as recipients of liver grafts. While the specific criteria for active listening for LT differ between countries and sometimes even between transplantation centers within a country, in many of them, the rule of ‘6 month’s abstinence’ prior to being listed is enforced and a relapse into alcohol consumption leads to delisting [18,19]. Notably, assessments prior to LT are to include a psychiatric and psychological evaluation and treatment, further underscoring the importance of adequate and ongoing psychiatric care for patients with alcohol-related liver disease. Interestingly, evidence suggests that post-LT follow-up may be more relevant than pre-transplant selection in patients with LT due to ALC [23,24]. Furthermore, while multiple measures aim to ensure just and transparent access to LT as a lifesaving procedure, a lack of evidence for sex to exert a significant effect on the likelihood of registration, transplantation and post-LT outcomes persists. However, previous data have shown that females are less often correctly identified for needing transplantation [25,26,27,28].

The possible disadvantage of females in the transplant allocation process has caused debate on how to best manage and reduce the stated inequality [29]. However, knowledge on what may cause this phenomenon and its dramatic implications is still lacking. Previous literature on the subject has, however, omitted the detailed analysis of alcohol-induced liver diseases in Europe. Thus, this study aimed to investigate the sub-group of patients with ALC, using the most recent data within the Eurotransplant region, for possible sex disparities in allocation to LT and respective outcomes in the context of specific epidemiologic characteristics of ALC.

## 2. Materials and Methods

In this study, all individuals listed for liver transplantation due to alcohol-related cirrhosis between 2010 and 2019 in the seven Eurotransplant countries (Austria, Belgium and Luxembourg acting in direct collaboration, and Croatia, Germany, Hungary, The Netherlands and Slovenia) were included. Access to data was granted by Eurotransplant. For every registration (or re-registration, respectively), during this time period, information on month/year of registration, age of the individual at time of registration, country of registration and the four types of outcomes (i.e., transplanted, removed, died on the waiting list or still on waiting list) and month/year of outcome event was available. Removal from the waiting list occurred due to the following reasons: transplanted outside of Eurotransplant, recovered recipient, recipient unfit for transplantation, wrong listing/administrative error and others.

The distribution of sex and type of outcome was described by frequency tables and calculating risk ratios and corresponding 95% confidence intervals. Mean age grouped by sex was calculated for the four outcomes.

Cumulative incidence was estimated by Fine–Gray competing risk models for outcomes (transplanted, died on waiting list, removed from waiting list) in an effort to take competing risk by the other two major outcomes into account. Sex, age at event and country were included as independent variables. The effect of sex is shown by cumulative incidence functions for females and males based on the regression model for a fictitious patient with mean age of all patients (i.e., 55.9 years) and German citizenship.

The study was exploratory in nature and, therefore, no adjustments for multiple tests were performed. The analysis was performed with SAS version 9.4 (SAS Institute Inc., Cary, NC, USA).

## 3. Results

### 3.1. Overview

For the included timespan between 2010 and 2019, 6182 listings of individual patients registered for a liver transplant due to alcohol-related liver cirrhosis were recorded. Out of all included cases, 349 individuals had been removed from the waiting list due to various reasons but were re-registered subsequently.

### 3.2. Sex Differences in Registration and Outcomes

A marked sex disequilibrium between females and males was found for registration totals and the four types of outcomes (see Section 2): female individuals accounted for 24.8% (*n* = 1532) of all registrations and for 20.4% (*n* = 684) out of all 3361 transplantations. In total, 27.0% (*n* = 385) of a total number of 1425 deaths on the waiting list were female patients, and 33.6% (*n* = 160) out of the 476 individuals who remained on the waiting list were female (see Table S1). Germany provided the largest share (62.8%) and Slovenia (1.8%) the smallest share out of all the seven countries that were included in this study (see Table S2).

#### 3.2.1. Successful Liver Transplantation

Risk ratios for different outcomes (i.e., transplantation and removal from waiting list) by sex were calculated for the Eurotransplant region as a whole and for each country separately. This analysis, when competing risk by other events was ignored, revealed that (1) the likelihood for females to receive a liver graft was lower than for males (risk ratio (RR): 0.78; 95% confidence interval (CI): 0.73–0.82) across the whole Eurotransplant region and, (2) a similar trend could be observed when each country was analyzed separately. However, the association of sex and the likelihood of receiving a liver graft only showed significant results for Belgium, Croatia and Germany, but not for Austria, Hungary and Slovenia.

**Figure 1:**
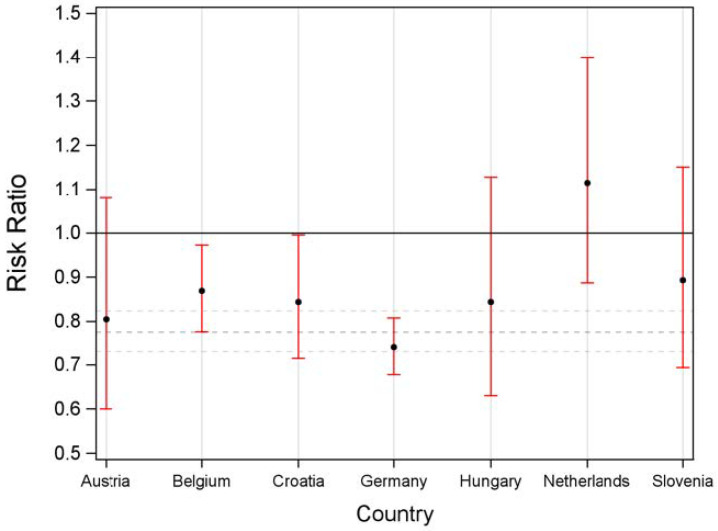
Risk ratio for transplantation depending on sex by country.

The Netherlands was the only country (out of all participating Eurotransplant countries) in which a higher relative share of female transplanted individuals (RR: 1.12; CI: 0.89–1.40) was observed; however, it was not statistically significant (Figure 1).

#### 3.2.2. Removal from Waiting List

While the majority (43.8%) of the cases were removed from the waiting list without a further specified reason (classified as “removed—other” by the Eurotransplant network), 21.4% were removed due to the recipient being unfit for transplantation (i.e., negative outcome) and 34.0% were removed as recipients recovered (i.e., positive outcome); 0.54% were removed due to wrong listing, administrative error or (0.22%) because they were transplanted outside the Eurotransplant region.

**Figure 2:**
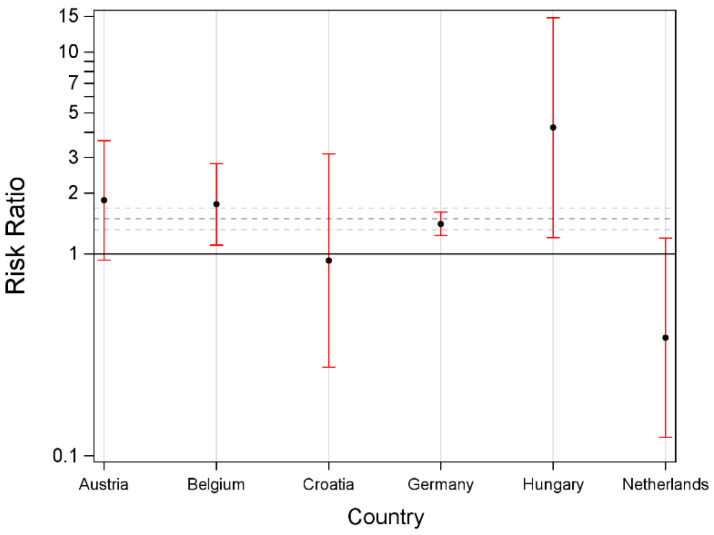
Risk ratio for being removed from the waiting list depending on sex by country.

Concurrent with the generally lower likelihood for females to receive a liver graft, the likelihood of being removed from the waiting list (irrespective of the reported reason) was also higher for females than males. When analyzing individual countries, this association was shown to be significant in Belgium, Germany and Hungary, but not in Croatia, Austria and the Netherlands. No data were available from Slovenia (Figure 2).

#### 3.2.3. Died on Waiting List

**Figure 3:**
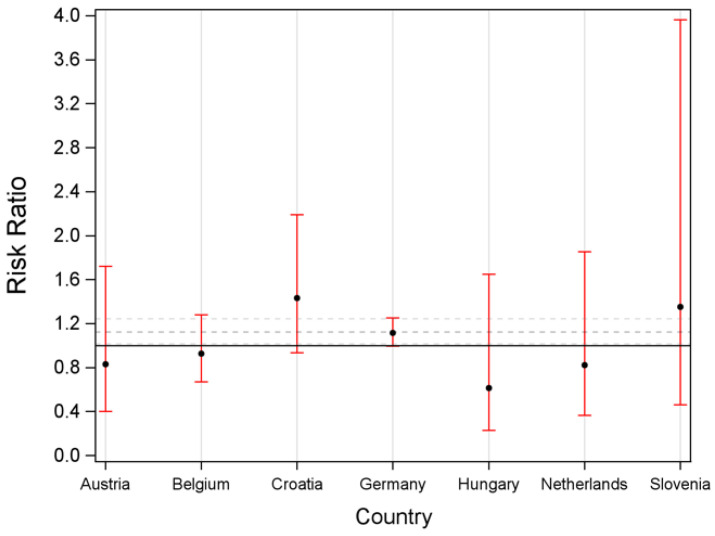
Risk ratio for having died while on the waiting list depending on sex by country.

Risk ratios for dying on the waiting list were borderline significant (RR: 1.12; CI 1.02–1.24) with slightly increased risk for females but inconsistent between countries (Figure 3).

#### 3.2.4. Age Differences between the Sexes

**Figure 4:**
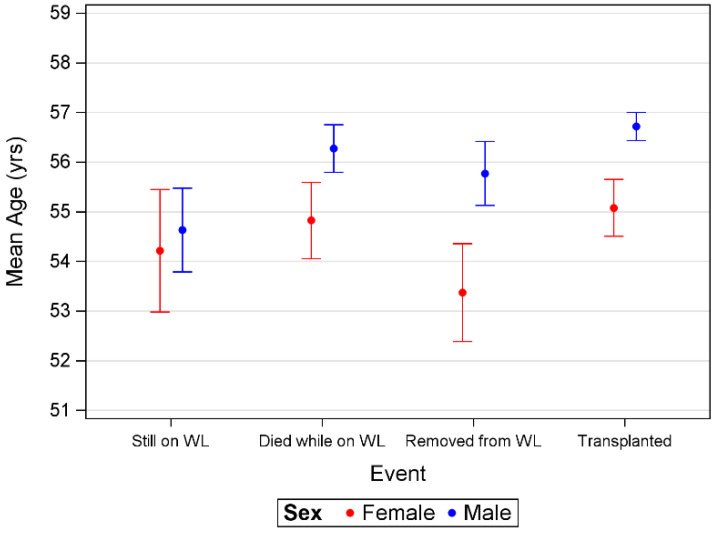
Mean age by sex for those patients still on waiting list (still on WL), those who died on waiting list (died while on WL), those removed from WL (removed from WL) and those transplanted (transplanted).

Significant differences in the mean age between females and males were observed: female individuals were significantly younger (mean age: 54.6; CI: 54.2–54.9) than males (mean age: 56.4; CI: 56.1–56.6) when enlisted for transplantation. Concurrently, female individuals who died on the waiting list (54.8; 54.1–55.6) or were transplanted (55.1; 54.5–55.7) were significantly younger than their male counterparts (56.3; 55.8–56.8/56.7; 56.4–57.0) (Figure 4).

### 3.3. Cumulative Incidences

**Figure 5:**
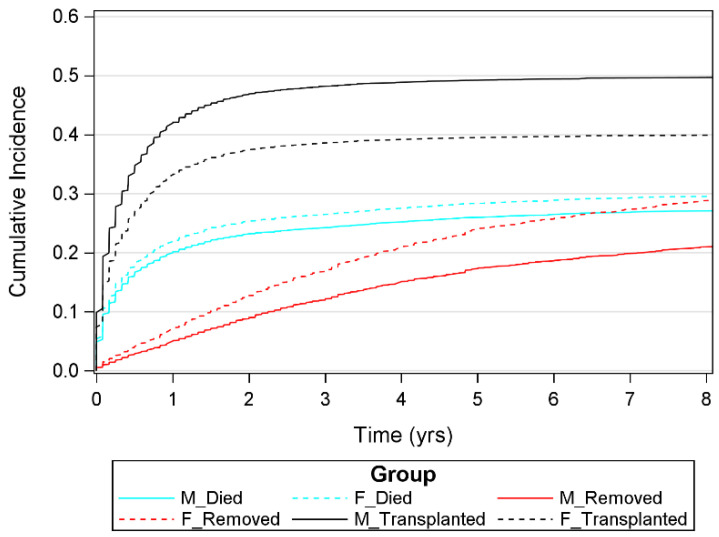
Cumulative incidence per year for males (M) and females (F) and the three outcomes examined (died while on waiting list, removed from waiting list and transplanted).

Survival time regression models accounting for competing risks between the outcomes, transplantation, removal from and death on waiting list were calculated. The Fine–Gray competing risk model revealed, concurrent to the analyses that had ignored competing risks, that females were less likely to receive an LT (hazard ratio (HR) 0.741) but more likely to be removed from the waiting list (HR 1.443) compared to males. Importantly, no significant effect of sex on cumulative incidences in females and males dying on the waiting list were revealed in the competing risk model. The effect of country was significant in all three models; the effect of age was borderline significant (*p* = 0.022) for the event transplanted only. See Figure 5 for the survival curves of the competing risk model.

## 4. Discussion

Our analysis revealed significant sex differences in registrations for LT due to alcohol-induced liver cirrhosis and in the subsequent outcomes in the Eurotransplant region. Specifically, our data indicated that the majority of registrations for liver transplantation (LT) due to alcohol-related liver cirrhosis (ALC) occurred in male patients. Furthermore, data showed that male patients registered for LT due to ALC were more likely to receive a liver graft, while their female counterparts were at higher risk of being removed from the waiting list and—at least in some countries—also of dying while being on the waiting list.

### 4.1. Sex Disparities in LT Registrations

The observed sex ratio of registrations for LT due to ALC in the present study (male to female ratio 3.0) was only partially in agreement with the sex difference for ALC according to the Global Burden of Disease Study that reported a male–female ratio up to 2.3 [2]. We suggest that the observed sex difference in registration for LT due to ALC should not be interpreted as a consequence of the sex-specific prevalence rates of ALC alone. Instead, other mediating factors in this association should be considered. Corresponding to the sex ratio in registrations for LT due to ALC, the male to female ratio in prevalence rates for alcohol use disorder was reported to reach 3.1 [2]. When comparing the stated findings to the male to female ratio for ALC of up to 2.3, one may assume that males with alcohol use disorder (AUD) are more likely to develop ALC. However, an alternative and rather overt explanation might be that males with ALC are more likely to be referred to transplantation centers, and registrations for LT are more commonly initiated in male patients than their female counterparts. This latter explanation is supported by findings describing persisting sex differences in perceived stigmata and, subsequently, treatment-seeking behaviors of patients with AUD [30,31,32]. Further corroborating this hypothesis, Lale et al. [33] reported that women are more likely to attribute AUD to someone’s “bad character” than men, resulting in pronounced stigmatization in females affected by this disease. Consequently, this may diminish the willingness of females to seek treatment and, thereby, elucidate why women who eventually enter treatment for their AUD are reported to be at already more advanced stages of the disease [31].

It seems likely that a complex interplay of various factors is responsible for the pronounced gender gap in LT registrations due to ALC. However, it cannot be ruled out that systemic and personal biases may play into the observed male-dominated prevalence ratio in LT registrations. This consideration is supported by an analysis of patients referred to a transplant center and evaluated for LT due to ALC by McElroy et al., in which men were revealed to be 95% more likely to be listed for LT than women [28]. As possible contributing factors, the authors suggested a higher prevalence of psychiatric comorbidities in females and hypothesized current screening protocols to be insufficient to identify alcohol abuse in women [28]. While previous studies have indicated existing gender and ethnicity disparities in LT [34,35,36,37,38], the relevance of the stated disparities in the context of ALC had not been assessed previously, resulting in the need for further research efforts.

### 4.2. Sex Differences in LT Outcomes

Our data revealed that the likelihood for females enlisted for LT due to ALC to eventually receive a liver graft was lower than for males (RR: 0.78) across the whole Eurotransplant region. Conversely, the likelihood of females being removed from the waiting list or dying while on the waiting list was also higher than for males. The reported differences, when analyzing the countries separately, were most likely due to the small number of observations in some of the countries (i.e., Austria, Hungary and Slovenia).

In contrast to the observed sex difference in registrations, the influence of sex on outcome metrics after listing for LT has been extensively discussed in previous research [29,39,40,41], along with a potential influence of ethnicity [25,26,27]. In an analysis of approximately 45,000 LT registrations, Moylan et al. concluded that even after introducing the model of end-stage liver disease (MELD) score as a selection criterion, the sex of the recipient remained a relevant factor associated with executed transplantation, while ethnicity was not [27]. Indeed, evidence suggests that sex disparities have become even more pronounced since the introduction of the MELD score and the stated difference has shown to be particularly pronounced in the sub-group of severely ill patients [42]. However, previous findings have been based on LT registration data irrespective of indication (i.e., including LT registrations due to other reasons than alcohol-induced liver cirrhosis). In contrast, the present study revealed similar data in the specific group of patients listed for LT due to ALC.

Although a comprehensive model explaining this difference is still lacking, some factors have been suggested in previous literature: First, the on average higher organ weight and increased organ size of donor organs from predominantly male donors are causing a mismatch between male donor organ weight and the weight of potential female organ receivers, further aggravating the shortage of donor organs. Secondly, the primary mechanism of prioritizing those patients on the waiting list—the model of end-stage liver disease (MELD) score—is being discussed as a potentially biased approach that disadvantages females [43,44]. Initially developed to predict mortality in patients with end-stage liver diseases, it is determined by the patient’s serum creatinine and bilirubin levels as well as the international normalized ratio (INR). In some areas, e.g., the United Network for Organ Sharing (UNOS) in the United States, an extended MELD score (MELD-Na) that also takes serum sodium levels into account is used. Notably, the MELD and MELD-Na scores were both reported to underestimate the severity of ALC in females; Cholongitas et al. [45] reported that females presented with significantly lower creatinine levels at an equally as compromised renal function as males. Subsequently, lower MELD scores in females led to a disadvantageous waiting list ranking. This systemic bias is hypothesized to be caused by a difference in body composition. As women tend to have less muscle mass than men and, thus, lower creatinine levels, the level of renal dysfunction may not be adequately reflected by serum creatinine levels [25,39,45].

However, previous efforts to counteract this tendency with the introduction and modification of standard exceptions (SE) and non-standard exceptions (NSE) for cases with further relevant risk factors not sufficiently reflected by the MELD score resulted in further imbalances [29,41].

A further important finding revealed in our analysis was that females were significantly younger than males when being listed for LT due to ALC and, correspondingly, were younger when receiving a liver graft. While not directly comparable, similar findings were reported in a recent analysis of 8408 patients listed for LT due to acute liver failure [46]. Conversely, in a different analysis of 45,688 registrations for LT (independent of causative etiology), it was reported that women were older than men at the time of listing [27]. In the case of registrations for LT due to ALC—as analyzed in the present study—we hypothesized that the stated finding resulted from a difference in the perceived stigmatization of alcohol consumption and AUD in both sexes, as previously mentioned [30,31,32]. Furthermore, the recent literature has shown an increase in the prevalence of AUD in females [47,48]. Interestingly, the anxiolytic effect of alcohol is described to be of higher importance in females in contrast to male alcohol consumption patterns. Recent stress-inducing events (i.e., the global financial crisis, in 2008, as well as the increase in immigration and insecurities permeating the society since 2015) may have aggravated the difference in alcohol consumption as a stress-related coping mechanism. Further findings that suggest that stress reactions in females are more pronounced than in males corroborate this hypothesis [49,50]. Importantly, estrogen and progesterone have been implicated to be associated with a higher sensitivity not only for reward signals in the central nervous system, but also for negative consequences of alcohol consumption [51,52,53,54].

### 4.3. Limitations

A separate analysis of sex disparities in specific sub-groups sub-grouped by measures of illness severity (i.e., MELD score or other clinical features such as parameters of liver function or the presence of refractory ascites) or by regional differences at the transplantation center level was not possible due to limited data availability. In order to not exceed a feasible number of competing risks for the analysis, it was not differentiated between the specific reasons for removal from the waiting list (“Wrong listing/administrative error”, “Transplanted outside of Eurotransplant”, “Recovered recipient”, “Recipient unfit for transplantation” or “Other”). However, as displayed in Table S5, the specific reasons for removal from the waiting list were similarly distributed across both sexes. Another potential limitation is that despite relatively specific clinical features and diagnostic criteria for ALC, diagnostic identification patterns might differ between individual transplant centers or might have been changed over the observed period. It cannot be ruled out that some of the reported findings were—at least to some extent—affected by these differences. Furthermore, two-thirds of the patients were registered in Germany, somewhat biasing the overall results when the effect of ‘country’ is not considered. Additionally, the numbers were small for some countries, so that the power to detect a significant effect of sex differed between countries.

Nevertheless, we believe these limitations were outweighed by the size of the study population and the trans-national approach.

## 5. Conclusions

In conclusion, distinct sex disparities were observed in registration and outcome metrics in LT due to ALC within the Eurotransplant region. One interpretation is that female patients may be at a disadvantage during the allocation process for an LT. Furthermore, a possibly disproportionately low registration rate was observed. As psychiatric and psychological assessments are commonly an integral part of the allocation process, these findings are of significant clinical importance for those psychiatrists working in association with transplant centers. Although some mechanisms that potentially account for the described effect of sex were identified, feasible measures to recognize and counteract this phenomenon in the clinical setting have yet to be established. Therefore, further research should focus on identifying and evaluating such measures, ensuring maximum utility and equity in LT due to ALC. We hope that the analysis presented in this manuscript further highlights the importance of the gender gap in registrations for LTX and advances the care provided to persons diagnosed with AUD in need of an LTX.

## Data Availability

The data that support the findings of this study are available from Eurotransplant (Eurotransplant Data Management Department). Restrictions apply to the availability of these data, which were used under license for this study.

## Supplementary Materials

The following supporting information can be downloaded at https://www.mdpi.com/article/10.3390/jcm11133646/s1, Supplementary Table S1: Results of the Fine-Gray competing risk model examining the likelihood of being removed from the waiting list, Supplementary Table S2: Results of the Fine-Gray com-peting risk model examining the likelihood of receiving a liver transplant, Supplementary Table S3: Results of the Fine-Gray competing risk model examining the likelihood of dying while on the waiting list, Supplementary Table S4: Listings per Country per Sex, Supplementary Table S5:Events per sex, Supplementary Table S6: Included Parameters in the MELD score, Supplementary Formula: MELD-Score, Supplementary Figure S1: Flow chart of all patients (male and female) with ALC registered for LTX in Eurotransplant, Supplementary Figure S2: Flow chart of male patients with ALC registered for LTX in Eurotransplant, Supplementary Figure S3: Flow chart of female patients with ALC registered for LTX in Eurotransplant.
