# Supplementary material for: Sex Disparities in Outcome of Patients with Alcohol-Related Liver Cirrhosis within the Eurotransplant Network—A Competing Risk Analysis
Source: J Clin Med. 2022 Jun 24;11(13):3646. doi: 10.3390/jcm11133646 (PMC9267400; doi:10.3390/jcm11133646)
Supplement: Supplementary file 1 [file jcm-11-03646-s001.zip › jcm-1746124-supplementary.pdf]

Supplementary Table S1: Results of the Fine-Gray competing risk model examining the likelihood of being removed from the waiting list
reference country: Germany; reference gender: male

Analysis of Maximum Likelihood Estimates				
Parameter		Parameter Estimate	P-Value	Hazard Ratio
**Country**	**Austria**	-0,42456	0,0116	0,654
**Country**	**Belgium**	-0,92478	<.0001	0,397
**Country**	**Croatia**	-1,86097	<.0001	0,156
**Country**	**Hungary**	-1,10261	0,0011	0,332
**Country**	**Netherlands**	-0,14516	0,4214	0,865
**Country**	**Slovenia**	-2,04166	0,0004	0,13
**Sex**	**Female**	0,36684	<.0001	1,443
**Age**		-0,0076	0,0897	0,992

Supplementary Table S2: Results of the Fine-Gray competing risk model examining the likelihood of receiving a liver transplant
reference country: Germany; reference gender: male

Analysis of Maximum Likelihood Estimates				
Parameter		Parameter Estimate	P-Value	Hazard Ratio
**Country**	**Austria**	0,35393	<.0001	1,425
**Country**	**Belgium**	0,57572	<.0001	1,778
**Country**	**Croatia**	0,64097	<.0001	1,898
**Country**	**Hungary**	0,51136	<.0001	1,668
**Country**	**Netherlands**	0,42111	<.0001	1,524
**Country**	**Slovenia**	0,85591	<.0001	2,354
**Sex**	**Female**	-0,29918	<.0001	0,741
**Age**		0,00511	0,0218	1,005

Supplementary Table S3: Results of the Fine-Gray competing risk model examining the likelihood of dying while on the waiting list.
reference country: Germany; reference gender: male

Analysis of Maximum Likelihood Estimates				
Parameter		Parameter Estimate	P-Value	Hazard Ratio
Country	Austria	-0,44151	0,0012	0,643
Country	Belgium	-0,39953	<.0001	0,671
Country	Croatia	-0,57243	<.0001	0,564
Country	Hungary	-0,41329	0,0346	0,661
Country	Netherlands	-0,53357	0,0021	0,587
Country	Slovenia	-0,84558	0,0016	0,429
Sex	Female	0,1027	0,0847	1,108
Age		0,00378	0,2834	1,004

Supplementary Table S4: Listings per Country per Sex

N (%)	Country							
Sex	Austria	Belgium	Croatia	Germany	Hungary	Netherlands	Slovenia	Total
Female	47<br>14,4	229<br>26,0	105<br>16,8	1050<br>27,0	32<br>22,9	43<br>20,1	26<br>22,8	1532
Male	279<br>85,6	652<br>74,0	519<br>83,2	2833<br>73,0	108<br>77,1	171<br>79,9	88<br>77,2	4650
Total	326<br>5,3	881<br>14,3	624<br>10,1	3883<br>62,8	140<br>2,3	214<br>3,5	114<br>1,8	6182

Supplementary Table S5:Events per sex.
Still onWL: still on waiting list
Died: Died while on waiting list
Removed-Other: Other
Removed-Recipi: Recipient unfit for transplantation" or "Other").
Removed-Recove: Recovered recipient
Removed-Transp: Transplanted outside of Eurotransplant
Removed-Wrongl: Wrong listing/administrative error
Transpl: Transplanted

Event by sex			
	Sex		
Event	Female	Male	Total
**Died**	385	1040	1425
	27.02	72.98	
	25.13	22.37	
**Removed-Other**	143	260	403
	35.48	64.52	
	9.33	5.59	
**Removed-Recipi**	44	153	197
	22.34	77.66	
	2.87	3.29	
**Removed-Recove**	115	198	313
	36.74	63.26	
	7.51	4.26	
**Removed-Transp**	0	2	2
	0	100	
	0	0.04	
**Removed-Wrongl**	1	4	5
	20	80	
	0.07	0.09	
**Still onWL**	160	316	476
	33.61	66.39	
	10.44	6.8	
**Transpl**	684	2677	3361
	20.35	79.65	
	44.65	57.57	
**Total**	1532	4650	6182

Supplementary Table S6: Included Parameters in the MELD score

List of included parameters in MELD Score
Creatinine
Bilirubin
INR (International Normalized Ratio)

$$10 \times [(0.965 \times \ln Creatinine) + (0.378 \times \ln Bilirubin) + (1.12 \times \ln INR)] + 6.43$$

Supplementary Formula: MELD-Score

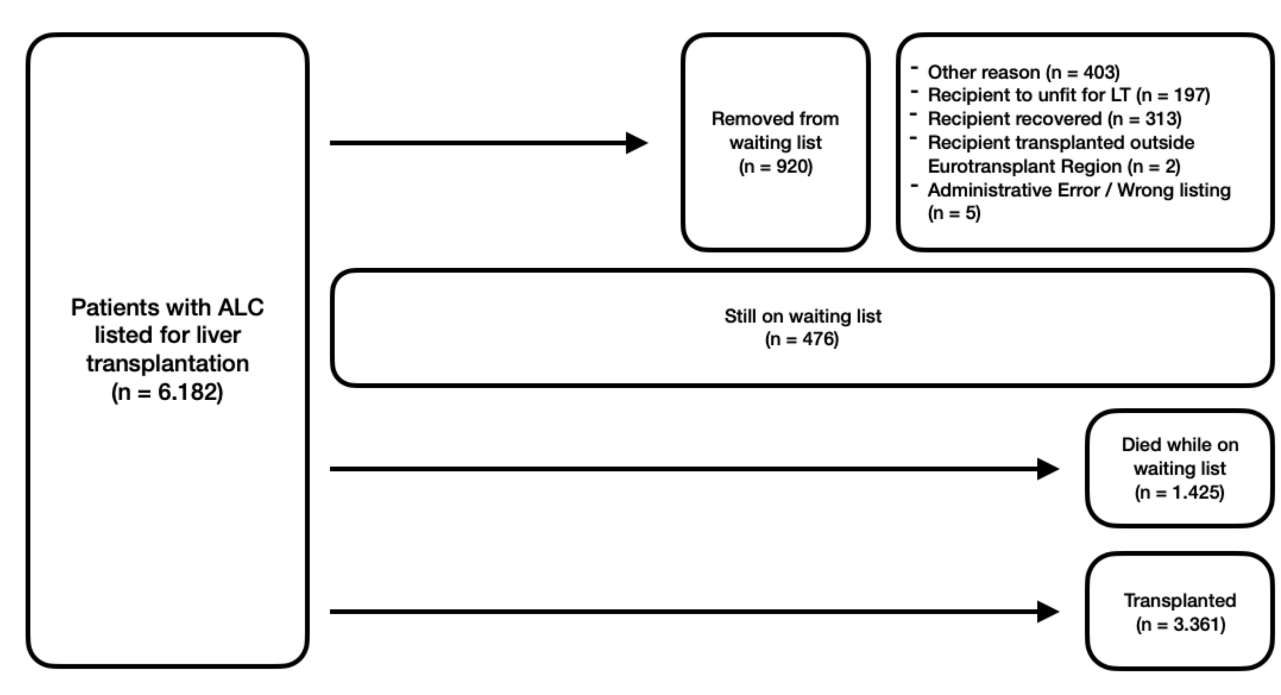

Supplementary Figure S1: Flow chart of all patients (male and female) with ALC registered for LTX in Eurotransplant

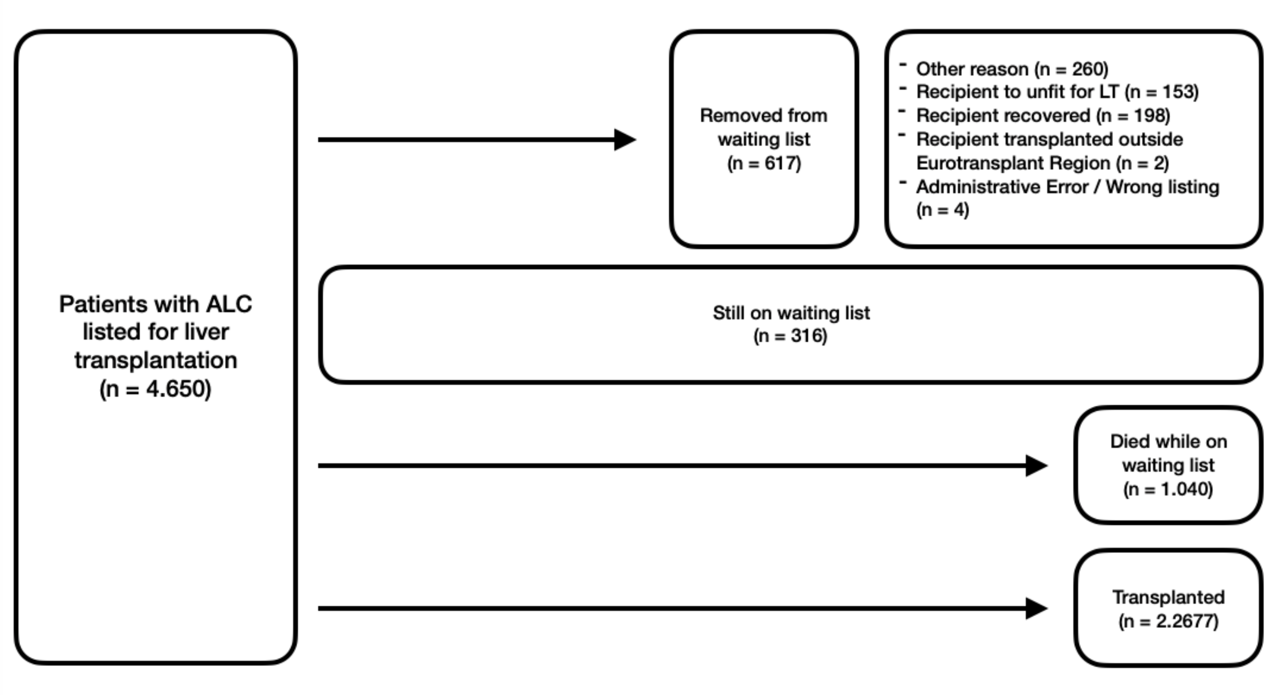

Supplementary Figure S2: Flow chart of male patients with ALC registered for LTX in Eurotransplant

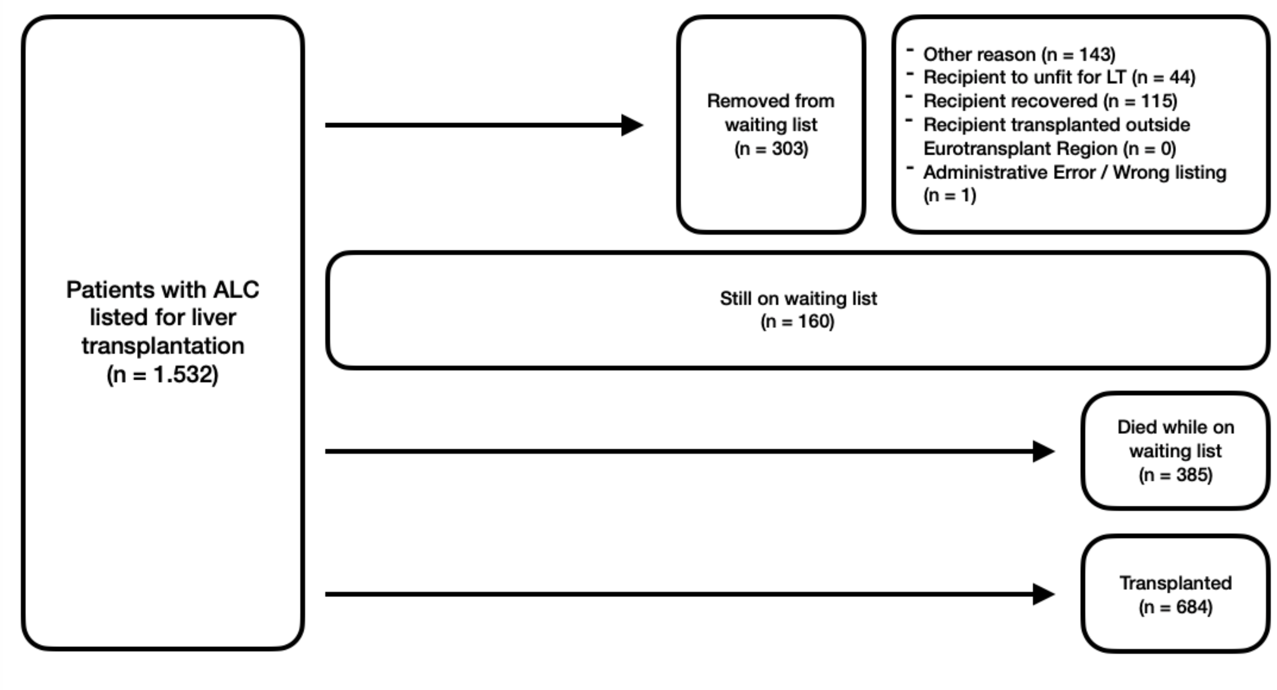

Supplementary Figure S3: Flow chart of female patients with ALC registered for LTX in Eurotransplant
